# Hydrophobia in South Wales

**Published:** 1902-06-14

**Authors:** 


### HYDROPHOBIA IN SOUTH WALES.

The recent occurrence of a case of hydrophobia in South Wales is a matter of some importance as showing how great is the uncertainty which attends even the most carefully directed efforts to stamp out a disease of this nature. In addition to this, the case itself is one of considerable interest in view of the long time which is stated to have intervened between the infection and the development of the symptoms. The patient, a man 63 years of age, was bitten in August, 1900, by a fox terrier, the bite being a severe one upon the exposed hand. The patient went to the Pasteur Institute, in Paris, and after 19 days' treatment returned home. He then felt quite well, and so remained until May 26, 1902, when he complained of pain in his arm. Symptoms of hydrophobia rapidly set in, and he died on May 28. The dog had shown some symptoms of rabies before it bit the patient, and was shot immediately afterwards.

